# Middle bile duct cancer with portal vein tumor thrombus

**DOI:** 10.1186/1477-7819-6-48

**Published:** 2008-05-10

**Authors:** Mitsugi Shimoda, Yukihiro Iso, Shigeki Tomita, Takahiro Fujimori, Koji Murakami, Tokihiko Sawada, Keiichi Kubota

**Affiliations:** 1 Department of Gastroenterological Surgery, Dokkyo University School of Medicine, Tochigi, Japan; 2 Department of Surgical Pathology, Dokkyo University School of Medicine, Tochigi, Japan; 3 P.E.T Center, Dokkyo University School of Medicine, Tochigi, Japan

## Abstract

**Background:**

Tumor thrombus in the portal vein is a common complication of hepatocellular carcinoma, but an extremely rare complication of common bile duct cancer.

**Case presentation:**

A 78-year-old woman was referred to our department because of jaundice. Laboratory data showed severe liver dysfunction with high serum levels of total bilirubin and CA19-9. Computed tomography showed lesions in the middle bile duct and main portal vein. FDG PET scan and 3D imaging showed hot spots in the same location as those revealed by CT. Under a diagnosis of middle bile duct cancer with portal vein tumor thrombus, the patient underwent surgery. At laparotomy, the main tumor was found to be located in the middle bile duct with a tumor thrombus, 2 cm in diameter, in the main portal vein. The patient underwent pancreatoduodenectomy with thrombectomy. Histological examination showed that this thrombus had the same histological features as those of the main bile duct cancer (poorly differentiated adenocarcinoma). The postoperative course was uneventful and the patient is doing well without any signs of recurrence 18 months after surgery.

**Conclusion:**

To our knowledge, this is the first report of successful resection of middle bile duct cancer with portal vein tumor thrombus.

## Background

Bile duct cancer (BDC), classified as cancer of the middle or distal bile duct, was the 6th leading cause of cancer-related death in Japan in 2004 [1]. Surgical resection offers the only chance for cure, and the outcome is highly dependent on surgical skill. In many patients, however, BDC is unresectable because of associated distant metastasis or involvement of the portal structures or main artery.

Portal vein tumor thrombus (PVTT) is sometime detected in patients with advanced hepatocellular carcinoma (HCC) or hilar cholangiocarcinoma (CCA) by preoperative imaging [2,3]. Although the bile duct is adjacent to the portal vein anatomically, BDC is rarely complicated by tumor thrombus in the main portal vein (PV). Here we present the first reported case of successfully resected middle BDC complicated by PVTT.

## Case presentation

**Figure 1:**
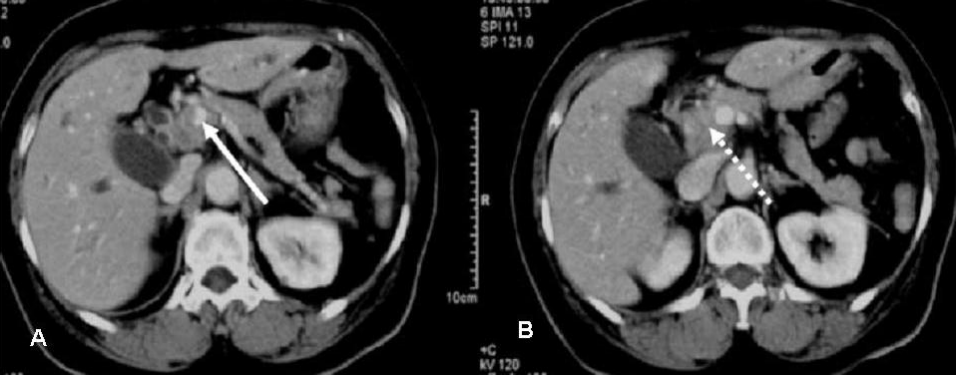
a and b. Computed tomography shows the PVTT (white arrow) and bile duct cancer (white dotted arrow).

**Figure 2:**
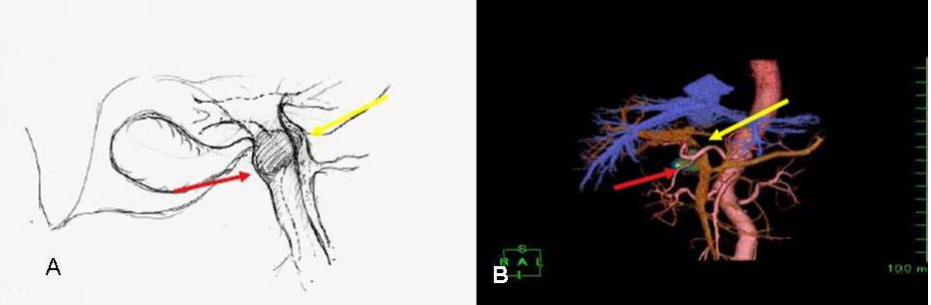
a and b. A schematic figure, main tumor shows red arrow in the main bile duct and portal vein shows yellow arrow. FDG pet scan with 3-D imaging shows hot spots in the main bile duct (red arrow) and main portal vein (yellow arrow).

**Figure 3:**
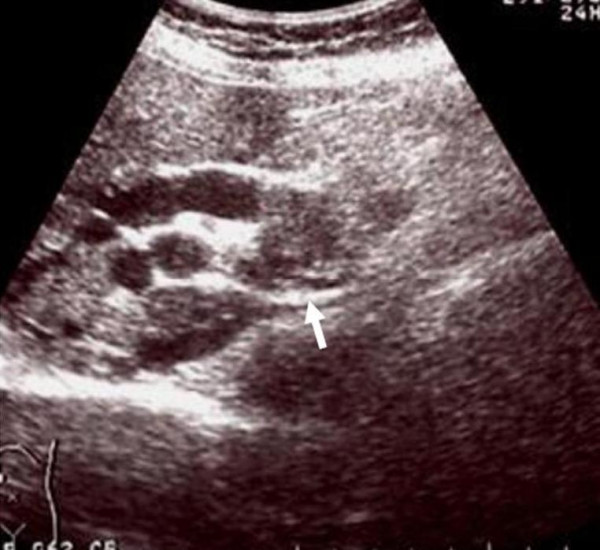
Intraoperative ultrasound clearly demonstrates the portal vein tumor thrombus.

**Figure 4:**
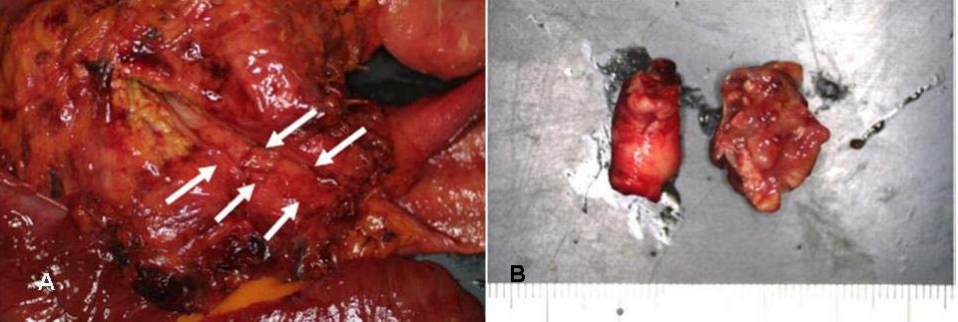
a and b. The main tumor, 3 cm in diameter, was located in the middle bile duct (a: white arrow) (Figure 3a) and the tumor thrombus (b), 2 cm in diameter, was present in the main portal vein (Figure 3b).

**Figure 5:**
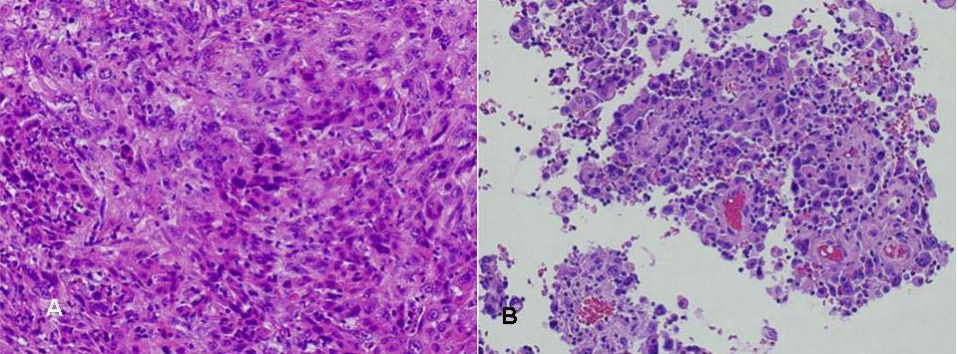
a and b. Histologically, both the bile duct cancer (Fig. a) and tumor thrombus (Fig. b) show the features of poorly differentiated adenocarcinoma.

A 78-year-old woman was admitted to our department with jaundice in July 2005. Laboratory data showed severe liver dysfunction with high serum levels of total bilirubin (7.2 mg/dl: normal less than 1.0 mg/dl) and CA19-9 (7600 U/ml: normal less than 30 U/ml). Computed tomography (CT) showed a mass in the middle bile duct and main PV (Figure 1a and 1b). Positron emission tomography with fluoro-2-deoxy-D-glucose (FDG PET) scan and 3D imaging showed hot spots in the main bile duct and main PV (Figure 2) and significant development of collateral vessels in the hepato-duodenal ligament. After reduction of serum total bilirubin, under a diagnosis of middle BDC with PVTT, the patient underwent surgery. At laparotomy, the main tumor was located in the middle bile duct, and a tumor thrombus 2 cm in diameter was present in the main PV. We performed pancreatoduodenectomy (PD) with extraction of the PVTT by PV incision, because it was unclear whether the cancer had invaded the PV. Intraoperative ultrasound clearly demonstrated the PVTT (Figure 3). Macroscopically, there was a circular lesion obstructing the bile duct, with a fragile tumor thrombus 2 cm in diameter (Figure 4a,b). Histological examination showed that bile duct cancer was a poorly differentiated adenocarcinoma, T4, N2, M0 and stage IV, according to the AJCC classification [4], and the thrombus showed the same histological features as those of the main tumor (Figure 5a,b).

The patient's postoperative course was uneventful. Adjuvant chemotherapy using Gemcitabine (1200 mg/day for 3 consecutive weeks with a rest in the 4^th ^week) was instituted 3 months after surgery. Currently, she is doing well without any signs of recurrence 18 months after surgery.

## Discussion

PVTT, which is occasionally observed in HCC, hilar CCA, pancreatic endocrine tumor and liver metastases from cancers of the gastrointestinal tract cancer including colorectal cancer, is caused by direct cancerous invasion into the PV and is associated with poor prognosis [2,3,5-8].

HCC shows a high incidence of PV invasion, and is reportedly found in 64.7% of patients at autopsy [2]. The incidence of PVTT is reported to be 5.3–15.4% in patients who undergo liver resection [5,6]. The median survival of patients with HCC and PVTT is only 2.4 months without treatment, whereas that in patients without PVTT is 24.4 months [9]. The hilar CCA also shows a high incidence of PVTT, and patients with this type show a slightly lower survival rate than patients with other types of CCA (intrahepatic CCA without portal vein thrombus) [3].

Between 1988 and 1997, 651 cases of BDC were resected in Japan with a 5-year survival rate of 23% for middle BDC and 32% for lower BDC [1]. However, there were no BDC cases complicated by PVTT. Generally, CT and abdominal ultrasound are helpful for preoperative diagnosis of PVTT [10,11]. In the present case, FDG-PET scan combined with CT clearly demonstrated the main lesion with PVTT. Development of collateral veins due to portal occlusion was also clearly visualized. Although PVTT is extremely rare in BDC, on the basis of these findings we diagnosed this portal occlusion not as cancer invasion but as PVTT.

Recently, FDG-PET scan has been widely adopted for diagnosis of pancreato-biliary malignancy and metastatic liver tumors, and is reported to be useful for detection of extrahepatic metastasis [12-17]. FDG-PET scan combined with CT will become a key examination for hepato-biliary pancreatic malignancies. Although PVTT due to other malignancies is caused by direct invasion of the PV and is usually connected to the main tumor, in the present case the connection between the BDC and PVTT was unclear and there was no clear direct invasion to the PV. Therefore, combined resection of the PV including the PVTT was not performed, and the PVTT was extracted by dividing the PV. Although histological examination was also unable to demonstrate invasion, it is likely that direct microscopic invasion to the PV would have been present, resulting in PVTT.

As mentioned above, the prognosis of malignancies associated with PVTT is usually poor. Our patient received standard adjuvant chemotherapy using gemcitabine, [18] and is currently doing well without any signs of recurrence 18 months after surgery.

## Conclusion

We have described the first reported case of BDC with PVTT, with special reference to the diagnostic utility of FDG-PET scan. Even if the condition is advanced, surgical treatment combined with adjuvant chemotherapy may be feasible in selected patients.

## Abbreviations

BDC: Bile duct cancer; PVTT: portal vein tumor thrombous; FDG-PET: Positron emission tomography with fluoro-2-deoxy-D-glucose; PV: Portal vein; HCC: hepatocellular carcinoma; CCA: cholangiocarcinoma, PD: pancreatoduodenectomy.

## Competing interests

The authors declare that they have no competing interests.

## Authors' contributions

MS preparation of the manuscript, concept and design and critically revising the manuscript. YI preparation of manuscript and concept and design, TS preparation of manuscript and concept and design, ST preparation of manuscript and concept and design, TF preparation of manuscript and concept and design, KM preparation of manuscript and concept and design, KK preparation of manuscript and concept and design. All authors read and approved final manuscript for publication.
